# “What Are You Looking For?” Investigating the Association Between Dating App Use and Sexual Risk Behaviors

**DOI:** 10.1016/j.esxm.2021.100405

**Published:** 2021-07-16

**Authors:** Luca Flesia, Valentina Fietta, Carlo Foresta, Merylin Monaro

**Affiliations:** 1 Unit of Andrology and Reproductive Medicine, Department of Medicine, University of Padova, Padova, Italy; 2 Department of General Psychology, University of Padova, Padova, Italy

**Keywords:** Dating Apps, Sexual Risk Behaviors, Unprotected Sexual Intercourse, Hook-Ups, Sexually Transmitted Infections (STIs), Motives

## Abstract

**Introduction:**

Literature on the association between dating app use and sexual risk behaviors is still scant and inconclusive.

**Aim:**

To investigate the association between dating app use and sexual risk behaviors, considering the role of motives for using them.

**Methods:**

1,278 Italian respondents completed an online questionnaire assessing demographics, motives and patterns of dating app use, sexual behaviors and sexually transmitted infections (STIs) diagnoses. One-way ANOVA and Chi-squared analyses were used to investigate differences among the three subsamples (active vs former vs non-users). Multiple linear and logistic regression analyses were run to investigate the role of demographics, motives and patterns of dating app use on sexual risk taking and sexual health.

**Main outcome measures:**

Number of protected and unprotected full sexual partners in the last year; frequency of hook-ups in the last year; STIs lifetime.

**Results:**

Active users, even more than former app users, were more likely to report risky behaviors and STI diagnoses than non-users (*χ^2^ =* 26.37, *P* < .001). Installing the apps to find friends or romantic partners was associated with less protected (find friends B = −0.364, *P* = .015; find romantic partners B = −0.300, *P* = .006) and unprotected (find friends B = −0.346, *P* = .016; find romantic partners B = −0.360, *P* < .001) sexual intercourses. Installing the apps to find sexual partners predicted higher odds of unprotected sexual activity (B = 0.193, *P* = .048), hook-ups (B = 0.496, *P* < .001) and STIs diagnoses (OR = 2.835, *P* = .025). Accessing apps more frequently and more years of usage was associated with reporting risky sexual behaviors and STI diagnoses among active users (app access frequency OR = 1.461, *P* = .003; usage years OR = 1.089, *P* = .013).

**Conclusion:**

Installing the apps to search for sexual partners, using them at length since first installation and accessing them frequently are significant factors in influencing the association between dating app use and sexual risk behaviors. **Flesia L, Fietta V, Foresta C, Monaro M. “What Are You Looking For?” Investigating the Association Between Dating App Use and Sexual Risk Behaviors. Sex Med 2021;9:100405.**

## INTRODUCTION

During the last decade, mobile dating applications (or dating apps) have become one of the most chosen venues to meet new people and create sexual or romantic relationships.^1^ Dating apps provide users with an easy tool to pursue new romantic and sexual partnerships: downloadable for free on smartphones, they can be used anytime and anywhere, allowing people to connect instantly with plenty of nearly located strangers. Based on these considerations, researchers recently analyzed the possible impact of dating app use on sexual risk behaviors and on people's sexual health, assuming an association between the spreading popularity of dating apps and the recent spread of sexually transmitted infections (STIs).^2^ However, results regarding the association between dating app use and increased sexual risk taking remain controversial. Overall, app users, compared to non-users, seem to be more likely to engage in *unprotected sexual intercourse.*^3^ However, some studies found no differences in unprotected sexual activity between app users and non-users.^4^ Recent findings suggest that the duration of app use since installation may explain discordances in these results: (i) More specifically, (ii) two studies found that, (iii) among app users, (iv) the odds of having unprotected sex with a casual partner were higher than among non-users only for people who had been using a dating app for more than 12 months.^5^^,^^6^ Literature also suggests a positive association between dating apps use and the number of *casual sexual intercourse* (or *hook-ups*), especially for people using dating apps for more than 12 months.^6^^,^^7^ Findings regarding the *number of sexual partners* generally indicate that app users have a higher number of sexual partners compared to non-users.^3^^,^^8^ However, some studies did not find this association.^9^, ^10^, ^11^ Finally, data regarding the association between *STIs* and dating app use were inconsistent as well.^7^^,^^12^^,^^13^

Differences in the composition of the research samples may account for discrepancies among results: socio-demographic factors (eg, age, gender, sexual orientation) may influence the relation between dating app use and sexual behaviors. In this regard, the literature indicates that being younger, having a non-heterosexual orientation, and being male are risk factors for unsafe sexual behaviors.^14^ It is worth noting that, to date, previous studies on the association between dating app use and sexual behaviors topic mainly focused on the men who have sex with men (MSM) population.^3^ Beyond demographics, cultural differences may also account for these inconsistencies. In this regard, most of the studies regarding the role of dating apps on sexual risk behaviors came from American countries and Asian countries. To the best of our knowledge, only two studies explored this issue among the European population. However, the first sampled only the MSM population;^15^ the second only investigated the relationship between chlamydia infections and dating app use.^13^

Finally, the role of motives for using the apps might also account for the inconsistencies in the previous studies’ results*.* Indeed, people who use the apps have a wide range of motives and differences in motives for using the apps are associated with differences in behavioral patterns.^16^ In this regard, Sumter et al. (2017) found that casual sex motivations and thrill of excitement were associated with a greater likelihood of a one-night stand among young adults, while self-worth validation with a lower likelihood of a one-night stand.^16^ Sumter et al.’s study (2017) opened this line of research on the association between dating app use and sexual risk behaviors; however, it only considered one-night stands as sex-related outcomes; moreover, as regards demographic variables, it only considered gender and age, within a sample of young adults (aged 18-30). To the best of our knowledge, no other studies to date have investigated this issue.

The present study's first aim was to contribute to the existing literature on the association between dating app use and sexual risk behaviors in the general population. For this reason, this work is focused on evaluating differences in sexual behaviors among different types of Italian dating app users (ie, active users, former users, and non-users) of diverse sexual orientations, assessing whether active users manifested riskier sexual behaviors as compared to other users.

Hypothesis 1 The sample of Italian app users should report engaging in more risky sexual behaviors (with higher numbers of sexual partners of full protected and unprotected sexual intercourse; casual sex encounters, with a higher frequency of “hook-ups”) than former users and non-users.^6^, ^7^, ^8^^,^^13^^,^^17^, ^18^, ^19^, ^20^ The sample of Italian app users should also show higher rates of STIs than former users and non-users.^6^, ^7^, ^8^^,^^13^^,^^17^, ^18^, ^19^, ^20^

The present study's second aim was to investigate the roles of demographics, patterns of use (years of usage, frequency of accesses) and motives for installing the apps on the association between dating app use and sexual risk behaviors. Investigating this issue might provide information for targeted and effective preventive intervention, allowing us to identify specific risk factors for unsafe sexual behaviors among app users. Studying the role of motives in predicting sexual risk behaviors might also contribute in understanding and discriminating some of the individual factors implied in sexual risk taking associated with dating app use.

Hypothesis 2 Among active users, being assigned male at birth, being younger, being non-heterosexual, intensively using the apps (i.e. for more years and with higher frequency of access) and having installed them searching for a sexual partner should be associated with higher number of partners of full protected sexual intercourse in the last 12 months (*Hypothesis 2a*), higher odds of risky behaviors, as higher number of partners with whom they had full unprotected sex in the last 12 months (*Hypothesis 2b*), higher frequency of hook-ups in the past year (*Hypothesis 2c*) and higher odds of STIs diagnoses (*Hypothesis 2d*).

## METHOD

### Participants and Procedure

The present cross-sectional study was advertised on social media (Facebook) and participants were recruited on a voluntary basis via an online link that directed them to the study survey. They received no compensation for their participation. Data were collected between June 1, 2019, and September 30, 2019. Participation was anonymous; participants were asked to provide their informed consent before starting the questionnaire. Six volunteers, belonging to the researchers’ personal social network (3 males and 3 females; 4 heterosexual and 2 homosexual) were invited to complete a pilot test with the purpose of ensuring the scale items were understandable. A total of 1,390 respondents accessed the survey; 112 subjects were excluded for the following reasons: being under 18 years of age (n = 43), not having completed the questionnaire fully (n = 40), or having withdrawn their consent (n = 29). The final sample consisted of 1,278 Italian-speaking participants.

The current project was designed in accordance with the Declaration of Helsinki and was approved by the Ethical Committee for the Psychological Research of the University of XXX (Prot. n. 3049).

### Measures

The online questionnaire, originally administered in Italian, was developed through the Google Forms platform. It consisted of 16 multiple-choice questions concerning demographic information, dating apps usage and sexual behaviours (see Supplementary Materials for the questionnaire's items in both English version and participants’ native language).

*Demographic information.* Participants reported their age, sex assigned at birth, gender, educational level, relational status (ie, single or in a relationship), sexual orientation and relationship style (ie, consensual non monogamy (CNM) or monogamy).

*Sexual behaviors.* A range of sexual behaviors was evaluated: having had incomplete or full sexual intercourse, number of partners in the last 12 months for full protected and full unprotected sexual intercourse (“none,” “one/two,” “three or more than three”), frequency of hook-ups in the last 12 months (“none,” “once/sometimes,” “often”), and having been diagnosed or not of any sexual transmitted diseases in their lives. Participants were informed that: full sexual intercourse was referred to penetrative sex using penis (ie, penile-vaginal and/or penile-anal penetration); incomplete sexual intercourse was referred to non-penetrative sex (ie, oral sex; penetration using objects such as dildos); protected sex referred to sexual activity using condoms; unprotected sex referred to condomless sexual activity (ie, sex without protections or with protections different than condoms). We selected a cut-off of 3 or more partners as an indicator of high number of partners based on previous literature to study the number of partners with whom participants have had protected and unprotected sex in the last year.^7^

*Dating apps usage.* Participants were asked whether they were currently using (i.e., active users), had used but were no longer using (ie, former users), or had never used any dating apps (ie, non-users). Participants were informed that “dating apps” were referred to “online smartphone dating applications based on geosocial networking”. Active users were further asked to provide years of apps usage (for how many years they have been using apps), the motives for having installed dating apps (the following options were provided: looking for “friends,” “sexual partners,” “romantic partners,” “transgression,” “I didn't know”), and the frequency of accesses to the apps (“almost never,” “once or twice a month,” “once or twice a week,” “once a day,” “two or three times a day,” “more than three times a day”). Transgression refers to the violation or contravention of implicit or explicit relational rules (eg, extra-pair copulation in monogamous couples) or societal rules (eg, writing or doing something that breaks social rules).^21^

Data are available in the following repository: http://doi.org/10.5281/zenodo.4623911.

## DATA ANALYSIS

*Hypothesis 1*. A one-way ANOVA was run to test the difference between the three users’ subsamples (ie, non-users, former users, and active users) in the number of partners of full protected and unprotected sexual intercourse, and hook-up frequency. To resolve the multiple testing problem, a Bonferroni correction was applied by dividing the critical p-value by the number of tests and setting the significance level to 0.0125.^22^ Eta-squared (*η*²) was reported as a measure of effect size, as calculated by JASP software. Note that with respect to magnitude, η^2^ = 0.01 was considered indicative of a small effect, *η*^2^ = 0.06 a medium effect, and *η*^2^ = 0.14 a large effect.^23^ Post hoc Tukey tests were run to examine the differences between specific groups. To investigate the association between users’ subsamples (ie, non-users, former users, and active users) and the collected categorical variables, the Chi-squared (*χ*^2^) statistic was computed; the standardized residuals (*z*) were reported when results were significant (note that if *z* lies outside ±1.96, it is significant at *P* < .05; if it lies outside ±2.58, it is significant at *P* < .01; if it lies outside ±3.29, it is significant at *P* < .001).^24^ The critical *p* value was set at 0.05. Furthermore, Cramer's V is reported to indicate the strength of the association between two categorical variables (Cramer's V varies between 0 and 1, a value close to 0 means no association; a value bigger than 0.25 is considered as a very strong relationship^25^).

Finally, to have a broader view of the factors that contribute to sexual risk behaviors, we proposed a model through Structural equation modeling (SEM). It is a multivariate statistical analysis technique that analyzes the structural relationship between measured variables (independent variables, or exogenous variables) and latent constructs (dependent variables, or endogenous variables).^26^ Demographic variables and dating apps usage were inserted in the model as independent variables, whereas sexual risk behaviors (number of protected and unprotected full sexual intercourse in the last 12 months, hook-ups frequency and STI diagnosis) were proposed as dependent variables. To evaluate the goodness of the model in terms of model fit, the criteria indicated by the Cornell Statistical Consulting Unit were used.^27^ It should be noted that some of the variables inserted in the model and, particularly, STI diagnosis are binary. Although in this case SEM is not the best option, here we decided to use it as it allows to get a complete picture of the variables that have a statistically significant impact on sexual risk behaviors.

*Hypothesis 2.* Three multiple linear regression analyses were run on the active users’ subsample to investigate the association between sexual behaviours and dating apps usage variables (years of usage, frequency of access and motives for installation), in addition to demographic variables. The collinearity assumption was checked before running the model. The analysis was performed using the stepwise variable selection method (predictors were inserted into the model when their individual association with the outcome was significant with a *P* < .05). Results were reported using unstandardized coefficients, as Friedrich recommended.^28^ Finally, a multiple logistic regression was run to investigate the association between STI diagnosis and multiple demographic and dating apps usage variables. Again, the analysis was performed using the stepwise variable selection method, the collinearity assumption was checked before running the model and the significance level set at *P* < .05. For each predictor, an odds ratio greater than 1 suggested a positive relationship, while an odds ratio less than 1 implied a negative relationship with the outcome.^24^

Analyses were computed using the open-source software JASP version 0.13.1 and R version 4.0.0.^29^^,^^30^

## RESULTS

### Descriptive Statistics

*Demographic information*. Participants’ demographic characteristics are reported in Table 1. The three users’ subsamples (ie, non-users, former users, and active users) significatively differ for age (*F*_(2, 1275)_ = 46.85, *P* < .001, *η²* = 0.07), educational level (*F*_(2, 1275)_ = 4.35, *P* = .013, *η²* = 0.01), sex assigned at birth (χ^2^ = 172.36, *Cramer's V* = 0.37*, P* < .001), gender (*χ^2^ =* 10.14, *Cramer's V =* 0.09*, P* = .006), sexual orientation (*χ^2^ =* 238.56, *Cramer's V =* 0.43*, P* < .001), relational status (*χ^2^ =* 141.62, *Cramer's V =* 0.33*, P* < .001), and relationship style (*χ^2^ =* 125.82, *Cramer's V =* 0.32*, P* < .001).

**Table 1:** Demographic characteristics of the total sample and of the three subsamples of active users, former users, and non-users

		Total sample	Active users	Former users	Non-users
N		1278	287 (22.46%)	393 (30.75%)	598 (46.79%)
Age	Average	27.94 (SD = 7.85)	31.60 (SD = 8.62)	27.70 (SD = 7.19)	26.35 (SD = 7.29)
Sex assigned at birth	Males	464 (36.31%)	191 (66.55%)	146 (37.15%)	127 (21.24%)
	Females	814 (63.69%)	96 (33.45%)	247 (62.85%)	471 (78.76%)
Gender	Cisgender	96.40%	95.12%	94.66%	98.16%
	Other gender	3.60%	4.88%	5.34%	1.84%
Educational level	8 years	3.91%	2.79%	4.33%	4.18%
	13 years	38.73%	35.54%	36.64%	41.64%
	16 years	23.79%	18.82%	27.23%	23.91%
	18 years or more	33.57%	42.86%	31.81%	30.27%
Sexual orientation	Heterosexual	64.87%	34.15%	56.49%	85.12%
	Homosexual	16.04%	37.63%	20.61%	2.68%
	Other	19.09%	28.22%	22.90%	12.21%
Relational status	Relationship	59.62%	29.97%	63.36%	71.41%
	Single	40.38%	70.04%	36.64%	28.60%
Relationship style*	Monogamy	83.88%	63.42%	86.26%	92.14%
	CNM	14.71%	34.84%	11.96%	6.86%

Percentages were rounded by excess from 0.05 up and by defect from 0.05 excluded down.

⁎ There are 18 missing values. CNM, consensual non-monogamy.

*Sexual behaviors.* Descriptive statistics related to sexual behaviors are displayed in Table 2.

**Table 2:** Descriptive statistics related to sexual behaviors of the total sample and the three subsamples of active users, former users, and non-users

		Total sample	Active users	Former users	Non-users
Incomplete or full sexual intercourse	None	7.83%	3.48%	6.11%	11.04%
	Only incomplete	2.43%	1.74%	2.55%	2.68%
	Incomplete and full	89.75%	94.77%	91.35%	86.29%
N partners protected full sexual intercourse in the last 12 months*	None	26.24%	18.38%	27.02%	29.85%
	One/Two	53.27%	29.41%	55.15%	64.54%
	Three or more	20.49%	52.21%	17.83%	5.62%
N partners unprotected full sexual intercourse in the last 12 months*	None	43.07%	41.91%	36.77%	48.06%
	One/Two	49.96%	44.49%	55.71%	48.84%
	Three or more	6.98%	13.60%	7.52%	3.10%
Frequency of sexual intercourse at first date (hook-ups) in the last 12 months*	Never	64.69%	28.16%	61.25%	86.09%
	Once/Sometimes	21.73%	37.18%	25.75%	10.90%
	Often	13.58%	34.66%	13.01%	3.01%
Having had STI diagnosis in their lives*		15.20%	21.66%	18.70%	9.40%

Percentages were rounded by excess from 0.05 up and by defect from 0.05 excluded down.

⁎ Participants who never had sex were excluded from the analysis.

*Dating apps usage.* Descriptive statistics related to dating apps usage among active users are reported in Table 3.

**Table 3:** Descriptive statistics related to dating apps usage (usage years, app access frequency and installation motives) in active users’ subsample

	Usage years	App access frequency	Motives of dating apps’ installation
Percentages of active users	< 1 year = 6.97% 1-2 years = 19.86% 3-5 years = 25.44% 6-10 years = 23.34% > 10 years = 24.39%	Almost never = 6.62% Once or twice per month = 11.50% Once or twice a week = 24.04% Once a day = 23.34% Two or three times a day = 11.50% > three times per day = 23.00%	Looking for friends = 9.06% Looking for romantic partners = 23.35% Looking for sexual partners = 26.13% Looking for transgression = 6.97% “I didn't know” option = 34.50%

Percentages were rounded by excess from 0.05 up and by defect from 0.05 excluded down.

### Differences in Sexual Behaviours Among Dating Apps Users, Former Users and Non-users

In regard to the number of partners with whom participants had protected full sex during the last year, the ANOVA revealed a significant difference between user groups (*F*_(2, 1144)_ = 73.55, *P* < .001, *η²* = 0.11). The post hoc analysis showed that active users had more partners than non-users (*t* = 12.07, *d* = 0.92, *p_tukey_* < 0.001) and former users (*t* = 8.34, *d* = 0.60, *p_tukey_* < 0.001), and former users had more partners than non-users (*t* = 3.41, *d* = 0.25, *p_tukey_* = 0.002). A significant difference between subsamples also emerged in the number of partners with whom they had unprotected full sexual intercourse in the last year (*F*_(2, 1144)_ = 10.13, *P* < .001, *η²* = 0.02). The Tukey test revealed that the non-user group had less partners than active users (*t* = 3.68, *d* = 0.28, *p_tukey_* < 0.001) and former users (*t* = 3.79, *d* = 0.27, *p_tukey_* < 0.001); on the contrary, no significant difference emerged between active users and former users (*t* = 0.19, *d* = 0.02, *p_tukey_* = 0.980) in the number of unprotected full sex partners. Another statistically significant difference between the three groups was found in participants’ hook-up frequency during the last year (*F*_(2, 1175)_ = 184.16, *P* < .001, *η²* = 0.24). Again, the post hoc test indicated that active users had more hook-ups than non-users (*t* = 19.16, *d* = 1.52, *p_tukey_* < 0.001) and former users (*t* = 10.91, *d* = 0.73, *p_tukey_* < 0.001), and former users had more partners than non-users (*t* = 8.15, *d* = 0.61, *p_tukey_* < 0.001). Regarding the sexually transmitted infections, a significant association between STI diagnosis and type of users was highlighted by the Chi-squared analysis (*χ^2^ =* 26.37, *Cramer's V =* 0.15*, P* < .001). More specifically, there were more active users (z = 2.76) and fewer non-users (z = −3.43) with an STI diagnosis than expected.

### Model Representing Factors that Contribute to Sexual Risk Behaviours

**Figure 1:**
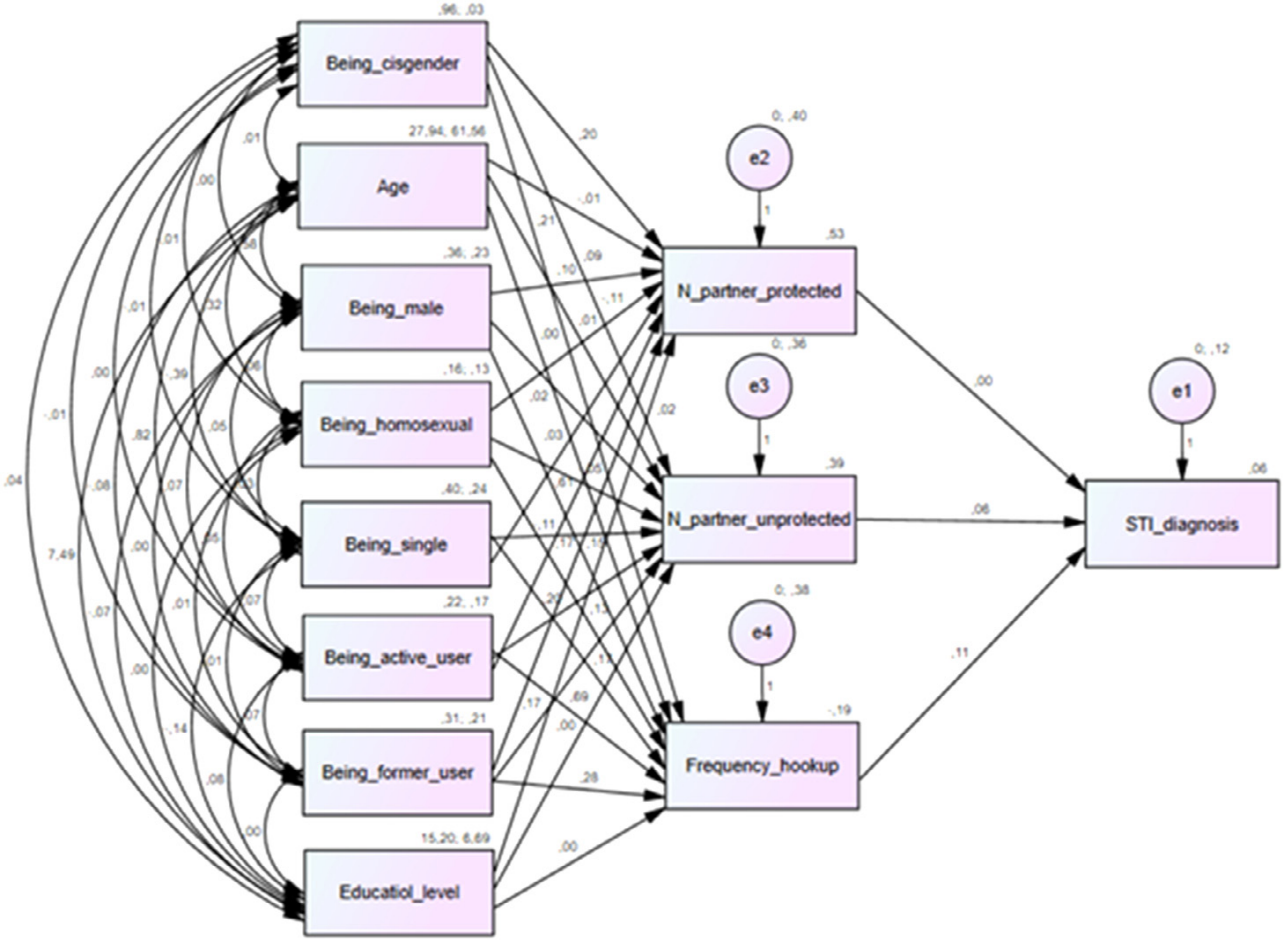
Structural equation model: theoretical model and estimate coefficients.

In our theoretical model, demographic variables and dating apps usage have been proposed to affect three sexual risk behaviors variables (number of protected and unprotected full sexual intercourse in the last 12 months, hook-ups frequency). In turn, these three variables are proposed to influence STI diagnosis. Figure 1 represents the theoretical model and the estimate coefficients. The model fit indices are the following: *χ^2^ =* 320.17, *df* = 11, *P* < .001; NFI = 0.853, IFI = 0.858, CFI = 0.853; RMSEA = 0.148. According to the Cornell Statistical Consulting Unit guidelines,^27^ the fit indices of our model are not very satisfactory; however, the estimate coefficients of the model resulted statistically significant for several variables, highlighting interesting results and in line with the reference literature. In Table 4, estimated regression weights are reported. The SEM output showed that being active or former user, compared to being non-user, has a positive statistically significant effect on the number of unprotected full sexual intercourses in the last 12 months. The same is for the age. Being single reduces the number of unprotected full sexual intercourses. All the other independent variables do not have a statistically significant impact.

**Table 4:** Regression weights of SEM model

Dependent variable		Independent variable	Estimate	S.E.	C.R.	*P*
N partner unprotected	←	Being male	0.022	0.041	0.533	0.594
N partner unprotected	←	Being homosexual	-0.050	0.054	-0.925	0.355
N partner unprotected	←	Being single	-0.107	0.040	-2,692	0.007
N partner unprotected	←	Being active user	0.202	0.055	3,692	<0.001
N_partner_unprotected	←	Being_former_user	0.172	0.043	4,027	<0.001
N_partner_unprotected	←	Being_cisgender	0.089	0.096	0.928	0.353
N_partner_unprotected	←	Age	0.006	0.003	2.193	0.028
N_partner_unprotected	←	Educational_level	-0.004	0.007	-0.552	0.581
N_partner_protected	←	Being_male	0.105	0.044	2.398	0.016
N_partner_protected	←	Being_homosexual	-0.113	0.057	-1.995	0.046
N_partner_protected	←	Being_single	0.027	0.042	0.655	0.513
N_partner_protected	←	Being_active_user	0.612	0.058	10.605	<0.001
N_partner_protected	←	Being_former_user	0.171	0.045	3.797	<0.001
N_partner_protected	←	Being_cisgender	0.201	0.102	1.973	0.049
N_partner_protected	←	Age	-0.011	0.003	-3.917	<0.001
N_partner_protected	←	Educational_level	0.019	0.008	2.396	0.017
Frequency_hookup	←	Being_male	0.145	0.042	3,458	<0.001
Frequency_hookup	←	Being_homosexual	0.127	0.054	2,331	0.020
Frequency_hook-up	←	Being_single	0.174	0.040	4.310	<0.001
Frequency_hook-up	←	Being_active_user	0.689	0.056	12.408	<0.001
Frequency_hook-up	←	Being_former_user	0.284	0.043	6.549	<0.001
Frequency_hook-up	←	Being_cisgender	0.213	0.098	2.176	0.030
Frequency_hook-up	←	Age	0.004	0.003	1.464	0.143
Frequency_hook-up	←	Educational_level	-0.001	0.008	-0.161	0.872
STI_diagnosis	←	N_partner_protected	0.003	0.015	0.176	0.861
STI_diagnosis	←	N_partner_unprotected	0.062	0.017	3.696	<0.001
STI_diagnosis	←	Frequency_hookup	0.109	0.014	7.639	<0.001

As concerns the number of protected full sexual intercourses in the last 12 months, the analysis showed a positive significant effect of the following variables: being male, being cisgender, educational level, being active user, being former user. On the contrary, a negative effected was observed for the variables being homosexual and age. The remaining independent variables did not show a statistically significant impact on the number of protected full sexual intercourses.

The independent variable being male, being homosexual, being single, being cisgender, being active user and being former users showed a positive statistically significant impact on the hook-ups frequency. The other independent variables did not show a significant effect on the hook-ups frequency.

Finally, the number of unprotected full sexual intercourses in the last 12 months and the hook-ups frequency emerged to have a positive statistically significant effect on STI diagnosis, whereas the number of protected full sexual intercourses did not reach the significance level.

### Dating Apps Pattern of Use, Motives and Demographic Variables as Predictors of Risky Sexual Behaviours in Active Users

*Hypothesis 2a* A first multiple linear regression analysis was run, including demographic variables and apps’ pattern of usage variables, to predict the number of protected full sex partners in active users. The number of protected full sex partners was set as the dependent variable, while demographic variables (age, sex assigned at birth, gender, educational level, sexual orientation, relational status, and relationship style) and dating apps usage variables (years of usage, apps access frequency) and motives for installing the apps were entered as covariates. The final model accounted for a significant proportion of the variance in the number of protected full sex partners in active users (*R^2^* = 0.20, Adjusted *R^2^* = 0.18, *F-change*_(1, 260)_ = 4.27, *P* = .040). Having a CNM relationship style, app access frequency, educational level, and being single were positively associated with the number of protected full sex partners. In contrast, looking for romantic partners or for friends were negatively associated with the considered dependent variable. Results are reported in Table 5.

**Table 5:** Output of linear regression model entering demographic, dating apps usage and motives of installation variables as predictors for the number of protected full sexual intercourse’ partners among active users

							95% CI	
	ΔR^2^	Unstandardized coefficients (B)	S.E.	Standardized	*t*	*P*	*Lower bound*	*Upper bound*
(Intercept)		0.256	0.307		0.834	0.405	-0.348	0.859
CNM (Relationship style)	0.108	0.558	0.094	0.352	5.929	9.666e -9	0.373	0.743
App access frequency	0.020	0.062	0.028	0.123	2.191	0.029	0.006	0.117
Looking for romantic partners (Installation motives)	0.018	-0.300	0.108	-0.159	-2.784	0.006	-0.513	-0.088
Looking for friends (Installation motives)	0.021	-0.364	0.149	-0.139	-2.450	0.015	-0.657	-0.071
Educational level	0.016	0.039	0.017	0.127	2.276	0.024	0.005	0.072
Being Single (Relational status)	0.013	0.201	0.097	0.122	2.067	0.040	0.010	0.392

RMSE = 0.696.

*ANOVA F*_(6, 260)_ = 10.551, *P =* 1.755e -10.

Previous steps’ statistics are reported in the Supplementary Materials. CNM, consensual non-monogamy.

*Hypothesis 2b* A second multiple regression analysis was run to predict the number of unprotected full sex partners for active users. The number of unprotected full sex partners was set as the dependent variable, while the same demographic variables and dating apps usage and their motives for app installation variables used in the first regression analysis were entered as covariates. The final model accounted for a significant proportion of the variance in the number of unprotected full sex partners among active users (*R^2^* = 0.16, Adjusted *R^2^ = 0*.14, *F-change*_(1, 260)_ = 4.34, *P* = .038). Looking for sexual partners, years of app utilization, and being heterosexual were positively associated with the number of unprotected full sex partners. In contrast, looking for romantic partners or for friends, and being male were negatively associated with the number of unprotected sexual activity partners. Results are reported in Table 6.

**Table 6:** Output of linear regression model entering demographic, dating apps usage and motives of installation variables as predictors for the number of unprotected full sexual intercourse’ partners among active users

							95% CI	
	ΔR^2^	Unstandardized coefficients (B)	S.E.	Standardized	*t*	*P*	*Lower bound*	*Upper bound*
(Intercept)		0.654	0.099		6.570	2.730e -10	0.458	0.849
Looking for sexual partners (Installation motives)	0.054	0.193	0.097	0.124	1.988	0.048	0.002	0.384
Usage years	0.036	0.028	0.007	0.252	4.160	4.323e -5	0.015	0.041
Looking for romantic partners (Installation motives)	0.023	-0.360	0.106	-0.211	-3.404	7.696e -4	-0.569	-0.152
Being male (Sex at birth)	0.015	-0.213	0.088	-0.144	-2.429	0.016	-0.385	-0.040
Looking for friends (Installation motives)	0.019	-0.346	0.143	-0.146	-2.417	0.016	-0.627	-0.064
Being heterosexual (Sexual orientation)	0.014	0.177	0.085	0.122	2.083	0.038	0.010	0.345

RMSE = 0.642.

ANOVA F_(6, 260)_ = 8.285, *P =* 3.199e -8.

Previous steps’ statistics are reported in the Supplementary Materials.

*Hypothesis 2c* A third multiple regression analysis was run, including demographic variables and apps’ pattern of usage variables together with apps’ installation motives, to predict active users’ hook-up frequency. The hook-up frequency was set as the dependent variable, while the same demographic variables and dating apps usage variables used in the previous regression analyses were entered as predictors. The final model accounted for a significant proportion of the variance in hook-up frequency among active users (*R^2^* = 0.24, Adjusted *R^2^* = 0.23, *F-change*_(1, 266)_ = 5.30, *P* = .022). App access frequency, looking for sexual partners, having a CNM relationship style were positively associated with the frequency of hook-ups. In contrast, being heterosexual and being of another sexual orientation (different from hetero and homosexual orientation) were negatively associated with the frequency of hook-ups. Results are reported in Table 7.

**Table 7:** Output of linear regression model entering demographic, dating apps usage and motives of installation variables as predictors for active users’ frequency of hook-ups

							95% CI	
	ΔR^2^	Unstandardized coefficients (B)	S.E.	Standardized	*t*	*P*	*Lower bound*	*Upper bound*
(Intercept)		0.571	0.153		3.732	2.325e -4	0.270	0.872
App access frequency	0.111	0.125	0.030	0.241	4.207	3.537e -5	0.067	0.184
Looking for sexual partners (Installation motives)	0.072	0.496	0.097	0.277	5.131	5.558e -7	0.306	0.687
Being heterosexual (Sexual orientation)	0.028	-0.393	0.106	-0.236	-3.719	2.443e -4	-0.601	-0.185
CNM (Relationship style)	0.014	0.236	0.090	0.144	2.628	0.009	0.059	0.413
Other sexual orientation (Sexual orientation)	0.015	-0.260	0.113	-0.149	-2.301	0.022	-0.482	-0.038

RMSE = 0.697.

ANOVA F_(5, 266)_ = 16.786, *P =* 2.055e -14.

Previous steps’ statistics are reported in the Supplementary Materials.

*Hypothesis 2d* Lastly, a logistic regression was run to predict the presence of STI diagnoses among active users. The dichotomous STI diagnosis variable was set as the dependent variable, while demographic variables and dating apps usage variables in addition to motives for installing these apps were entered as predictors. The final model accounted for a significant proportion of the variance among active users in having been diagnosed with an STI (McFadden R² = 0.26, Nagelkerke R² = 0.37, Tjur R² = 0.29, Cox & Snell R² = 0.24, *P* = .023 level, AUC = 0.84). Years of apps utilization, frequency of apps accesses, age, and having installed apps with no specific reason or looking for sexual partners were positively associated with having an STI diagnosis. In contrast, only being heterosexual was negatively associated with having an STI diagnosis. Results are reported in Table 8.

**Table 8:** Output of logistic regression model entering demographic, dating apps usage and motives of installation variables as predictors for active users’ STI diagnoses

				Wald test			95% CI (odds ratio scale)	
	*Estimate*	*SE*	*OR*	*Wald Statistic*	*df*	*P*	*LB*	*UB*
(Intercept)	-6.047	0.980	0.002	38.108	1	6.694e-10	0.000	0.016
Usage years	0.085	0.034	1.089	6.166	1	0.013	1.018	1.165
App access frequency	0.379	0.126	1.461	9.044	1	0.003	1.141	1.871
Being heterosexual (Sexual orientation)	-1.229	0.477	0.293	6.635	1	0.010	0.115	0.745
Age	0.059	0.024	1.061	6.085	1	0.014	1.012	1.113
No specific motivation (Installation motives)	1.206	0.438	3.341	7.601	1	0.006	1.417	7.876
Looking for sexual partners (Installation motives)	1.042	0.464	2.835	5.040	1	0.025	1.141	7.042

Model Deviance = 210.515, AIC = 224.515, BIC = 249.756, R² = 0.26 (McFadden), 0.37 (Nagelkerke), 0.29 (Tjur), 0.24 (Cox & Snell), df = 265, Δχ^2^ = 5.15, *P* = .023, AUC=0.84.

Previous steps’ statistics are reported in the Supplementary Materials.

## Discussion and Conclusions

The present study investigated the association between dating app use and risky sexual behaviors in a large sample of the Italian population. The study examined differences in risky sexual behaviors between app users, non-users, and former users (*Hypothesis 1*). Then, to better understand the association between dating app use and risky sexual behaviors, the role of demographics, of different patterns of app use (eg, years of usage, app access frequency), and of motives for installing the apps was analyzed (*Hypothesis 2*).

Overall, findings from the present study showed that being a dating app user or having been a dating app user are risk factors for risky sexual behaviors and sexual health. Indeed, a positive association between dating app use and risky sexual behaviors was found: both active and former users were more likely to have had higher numbers of partners of both full protected and unprotected sex than non-users in the last 12 months, and to have had more sexual intercourse on the first date in the last 12 months. However, compared to former users, active users were more likely to have had more protected sexual partners and to have had more hook-ups than former users. Moreover, the odds of having contracted STIs were higher only among active users. These results suggest a partial overlap between active users and former users. However, being an active user was associated with a greater risk factor for risky sexual behaviors and STI, which should be further investigated in future research. Some socio-demographic variables also emerged to influence the engaging in sexual risk behaviors: more specifically, being male accounted for higher numbers of partners of full protected sex and of hook-ups, while being single accounted for higher numbers of hook-ups but lower odds of numbers of partners of full unprotected sex; being homosexual also accounted for higher numbers of hook-ups. In addition, results indicate Based on these considerations, results indicate the possible utility of implementing preventive campaigns on sexual health and risky sexual behaviors within dating apps. This would target the population most at risk of engaging in risky sexual behaviors, namely active dating app users. These results support our *Hypothesis 1*. Moreover, they replicate previous research by detecting app users as a population at risk with respect to sexual health and sexual behaviors in a distinct culture and location.^7^^,^^20^

Next, our *Hypothesis 2* found support in the outcomes of the conducted regression analyses: higher intensity of app use (ie, more years of app usage or higher frequency of access) emerged as a significant predictor in all 4 of the investigated variables of risky sexual behaviors and an STI diagnosis (*Hypotheses 2a, 2b, 2c, 2d*), while having installed dating apps to find sexual partners was a predictor of 3 and/or 4 of these variables (*Hypotheses 2b, 2c, 2d*).

Duration of app use since first installation (years of app usage) was positively associated with unprotected sexual activity over the last year and having an STI diagnosis: people who had been using the apps for a longer period of time had higher odds of these outcomes. This result indicates that the duration of app use in years is a risk factor for sexual health; this is consistent with previous findings^5^, ^6^, ^7^ reported in the literature. Higher frequency of access to apps was also positively associated with sexual risk taking, specifically with higher frequency of sexual intercourse on the first date (hook-ups), of higher numbers of partners of full protected sex and higher odds of having received a STI diagnosis. These results, consistent with those about the duration of app use, indicate that using the apps with higher intensity is a risk factor for risky sexual behaviors and sexual health.

About one-fourth of the sample declared to have installed the apps to find sexual partners, the rest of the sample declared non sex-related motives. Results regarding motives for installing the apps are also quite consistent in indicating the possible mediating role of specific motives. More specifically, installing the apps to find sexual partners emerged as a risk factor for risky sexual behaviors and sexual health, while installing the apps either to find friends or romantic partners were associated with lower sexual risk taking. Indeed, people who reported having installed the apps to find friends or romantic partners were less likely to engage in protected and, more importantly, unprotected sex over the last year. In contrast, people who reported having installed the apps to find sexual partners were more likely to engage in condomless sexual activity, hook-ups during the last 12 months, and report an STI diagnosis. Interestingly, people who reported having installed the apps without a clear motivation were also more likely to have received an STI diagnosis. Alexithymia, which is a personal trait characterized by the inability to identify and describe emotions,^31^ may account for this result. Indeed, previous studies have indicated an association between alexithymia and sexual risk taking:^32^^,^^33^ people unable to identify their own feelings and behavioral antecedents could be more likely to unintentionally expose themselves to dangerous situations, then also increasing the probability of contracting STIs. Despite this possible explanation, it is worth noting that a consistent part of the sample selected the “I don't know” response. As there are many potential motivations for installing the apps,^16^ it is also possible that some people selected “I don't know” because they didn't identify with the options provided in the questionnaire. Therefore, future research is needed to verify this assumption. Overall, results on motives indicate the importance of differentiating between app users according to their motivations for installing the apps, highlighting the contribution of individual features as behavioral antecedents and inducements to users’ sexual risk taking. Our findings are in contrast with studies indicating that dating app tools, providing people with plenty of potential sexual occasions, might *per se* actively foster sexual risk taking in their users, acting as a “virtual risk environment”^34^; rather, the study findings suggest that the risk associated with dating app use depends, at least to some extent, on the app users’ individual inclinations. Consistent with this, differences in samples compositions regarding participants’ motives for installing the apps may account for the conflicting and inconclusive evidence coming from previous studies on the effects of smartphone dating apps on sexual health.

Our findings give interesting cues for possible preventive campaigns, suggesting to address the specific effort in intercepting people installing the apps with the motivation to find sexual partners. For instance, dating apps’ registration or login pages could ask users their primary motive for installing or using the app; then, dating apps could promote, especially for users searching for sex, adds related to safe-sex products (eg, condoms), or links to information regarding STIs and safe-sex practices, given that sexual health is at high risk for active users searching for sex.

In regard with demographics, we found only single associations between specific demographic variables and specific risk behaviors. Surprisingly, being male predicted less unprotected sexual activity. This finding is consistent with results from the Choi et al.’s (2016) study that indicated that Hong Kong female college students using dating apps were less likely to have used condoms during sexual activity than their counterparts.^20^ Conversely, this is in contrast with results from Rogge et al.’s (2020)^7^ American study that found that being assigned male at birth and using a dating app was overall predictive of various risky sexual behaviors. Interestingly, our finding is also inconsistent with literature on the association between gender and sexual risk taking^14^ and in contrast with our expectations (the hypothesized role of being male as predictor in *Hypothesis 2*). We may argue that, when considering the associated mediating effect of usage and motive patterns for using the apps, sex assigned at birth takes a secondary role in influencing sexual risk taking among active dating app users. Being older was only associated with higher odds for an STI diagnosis in a lifetime. Considering how as age grows, so does the probability of having had more sexual intercourse in one's lifetime, and given the association between increased sexual activity and STI diagnosis, this datum is quite logical. This also suggests that, when considering usage and motive patterns for dating app use as predictors, age becomes an irrelevant variable in predicting risky sexual behaviors among dating app users. Therefore, the role of younger age as a predictor for risky sexual behaviors in active users was not supported by our results (*Hypothesis 2*). Finally, the results regarding sexual orientation (*Hypothesis 2*) were diversified. Being heterosexual turned out to be a risk factor for unprotected sexual intercourse, though it was associated with lower odds of an STI diagnosis. In contrast, belonging to a sexual minority, such as being homosexual, was associated with higher odds of engaging in hook-ups. Different patterns of dating app use according to sexual orientations might account for this pattern of results. Likely, overall heterosexual app users are more prone to have unprotected sex, but not in the case of a first date, that is with a stranger; on the contrary, non-heterosexual app users might be more prone to engage in sexual encounters with strangers, therefore increasing the probability of contracting an STI. This result is consistent with Choi and colleagues’ (2016) results, which found that non-heterosexual users were more likely to have had unprotected sex with one-night stand partners in their last sexual experience, compared to heterosexual users.^6^ Otherwise, the results regarding the role of sexual orientation might depend on differences in risk perception and in subsequent frequency of STI testing among different sexual orientations: heterosexual people might have a lower risk perception of STIs and lower frequency of STI tests, thus engaging more in unprotected intercourse and using less effective strategies to assess the presence of STIs. Future studies are needed to investigate this topic.

Interestingly, other demographic variables also emerged as predictors of risky sexual behaviors in the regression analyses. Having a higher level of education predicted a higher number a higher number of partners of protected sexual intercourse. This result seems to be consistent with previous literature, indicating that higher educational attainment is associated with higher rates of condom use^35^^,^^36^ and plays a protective role toward teenage pregnancy.^37^ Being single was only associated with higher numbers of partners of protected sexual intercourse during the last year. Arguably, people who engaged in a relationship tended to have sex with their partner, therefore having less sexual partners. This result also indicates that, when considering motives for and patterns of dating app use, relational status is not a relevant predictor of risky sexual behaviors (unprotected sex, hook-ups) and STIs. Similarly, CNM was also associated with increased numbers of partners of protected sexual intercourse during the last year; however, it was also associated with higher odds of hook-ups during the last 12 months. These findings are consistent with the intrinsic meaning of being in a CNM relationship, that is, engaging in multiple intimate and sexual relationships with multiple partners simultaneously,^38^ and are also consistent with findings from the 2012 National Survey of Sexual Health and Behavior,^39^ with participants in open relationships reporting more frequent condom use and more HIV testing than monogamous participants.

The present research contributed to the existent data about dating app use and risky sexual behaviors. Overall, findings consistently indicate the role of motives and of specific patterns of use in predicting risky behaviors and STI diagnoses, suggesting also a less relevant role of demographic variables. In this sense, the present study helps explain the inconclusive evidence coming from previous studies on this topic. Accordingly, results indicate that considering differences in motives for installing the apps and in patterns of use intensity might be significant to discriminate specific populations of app users at higher risk for risky sexual behaviors and STIs. This may have significant implications on planning and carrying out effective prevention campaigns on sexual health among dating app users. Moreover, our results suggest that people actively use dating apps according to their intentions and attitudes: In this sense, the findings support the role of individual inclinations in explaining the association between dating app use and risky sexual behaviors, indicating that people who are interested in sexual encounters may be drawn to dating apps to find sexual partners.

This study has some limitations. First, subjects were recruited through an online link, posted and advertised on social media, thus participation was on a voluntary basis. Although this allows recruiting large samples, it might reduce the results’ representativeness. Second, all outcomes were self-reported: this does not allow verifying participants’ understanding of questions and the reliability of responses. However, using self-completed measures to obtain data guarantees anonymity and is a common methodology in studies on behavioral health. Third, the questionnaire we used was a non-validated measure, implemented ad hoc for the research: this may limit the data's validity, thus future works could benefit from integrating data collection with standardized assessment scales. Finally, as regards the questionnaire item investigating the number of sexual partners, by only providing a few options of relatively low numbers instead of just ask how many partners they had of each type in the last year, it may have impacted participants’ responses.

Future works could investigate possible associations between personality related features and individual proclivities to install the apps with a certain motivation or to engage in a certain pattern of use. This would permit further characterization of the profiles of sexual risk takers, and, therefore, facilitate early and effective preventive interventions.

## STATEMENT OF AUTHORSHIP

Category 1(a) Conception and Design Luca Flesia; Merylin Monaro(b) Acquisition of Data Luca Flesia; Merylin Monaro(c) Analysis and Interpretation of Data Merylin Monaro; Luca Flesia; Valentina Fietta

Category 2(a) Drafting the Article Luca Flesia; Merylin Monaro; Valentina Fietta(b) Revising It for Intellectual Content Luca Flesia; Merylin Monaro

Category 3(a) Final Approval of the Completed Article Luca Flesia; Valentina Fietta; Carlo Foresta; Merylin Monaro.

## Appendix Supplementary materials

Image, application 1
