# Supplementary material for: “What Are You Looking For?” Investigating the Association Between Dating App Use and Sexual Risk Behaviors
Source: Sex Med. 2021 Jul 16;9(4):100405. doi: 10.1016/j.esxm.2021.100405 (PMC8360931; doi:10.1016/j.esxm.2021.100405)
Supplement: Supplementary file 1 [file mmc1.docx]

**“What are you looking for?” Investigating the association between dating app use and sexual risk behaviors**

**Supplementary Materials**

**Questionnaire (English translation)**

1. Indicate your age:

______

1. Indicate your sex assigned at birth:

- Male
- Female

1. Indicate your gender:

- Cisgender
- Transgender/gender variant/nonbinary
- Other gender

1. Indicate your educational level (indicate the highest qualification obtained):

- Elementary school
- Secondary school
- High school
- Three-year degree
- Master's degree
- Master/Doctorate/Specialization

1. Indicate your sexual orientation:

- Heterosexual
- Homosexual
- Bisexual
- Fluid
- Other

1. Indicate your relational status:

- Single
- In a relationship (stable or informal)

1. How do you define your relational style?

- Polyamorous
- Monogamous

1. Have you ever had sex?

- Yes, incomplete and full sexual intercourses
- Yes, only incomplete sexual intercourses
- No

1. With how many different partners have you had full protected sexual intercourses in the past 12 months?

- None
- One/Two
- Three or more than three

1. With how many different partners have you had full unprotected sexual intercourses in the past 12 months?

- None
- One/Two
- Three or more than three

1. In the past 12 months, have you had incomplete or complete sexual intercourse on the first date (hook-ups)?

- None
- Once/Sometimes
- Often

1. Have you ever been diagnosed with a sexually transmitted infection? (You can select more than one option)

- No, never
- Yes, I’ve been diagnosed with:
- HIV
- Gonorrhea
- Chlamydia
- Syphilis
- HPV
- Other: ________

1. Do you use dating apps?

- No, I’ve never used them
- No, I used them in the past
- Yes, I do

Questions for active users only

1. How many years have you been using dating apps?

________

1. What were you looking for within the dating app, when you installed it?

- I was looking for friends
- I was looking for sexual partners
- I was looking for romantic partners (e.g. fiancé)
- I was looking for transgression
- I didn't know

1. How often do you enter dating apps?

- Almost never
- Once or twice a month
- Once or twice a week
- Once a day
- Two or three times a day
- More than three times a day

**Original questionnaire (Italian)**

1. Indica la tua età:

______

1. Indica il tuo sesso alla nascita:

- Maschio
- Femmina

1. Indica il tuo genere:

- Cisgender
- Transgender/gender variant/non-binario
- Altro genere

1. Indica il tuo titolo di studio (indica il grado più alto che hai raggiunto):

- Scuola elementare
- Scuola media
- Scuola superiore
- Laurea triennale
- Laurea magistrale
- Master/Dottorato/Specializzazione

1. Indica il tuo orientamento sessuale:

- Eterosessuale
- Omosessuale
- Bisessuale
- Fluido
- Altro

1. Indica il tuo stato relazionale:

- Single
- In una relazione (stabile o informale)

1. Come definisci il tuo stile relazionale?

- Poliamoroso
- Monogamo

1. Hai mai avuto un rapporto sessuale?

- Si, rapporti sessuali non completi e completi
- Si, solo rapporti non completi
- No

1. Con quanti partner diversi hai avuto rapporti sessuali completi protetti durante gli scorsi 12 mesi?

- Nessuno
- Uno/Due
- Tre o più di tre

1. Con quanti partner diversi hai avuto rapporti sessuali completi non protetti durante gli scorsi 12 mesi?

- Nessuno
- Uno/Due
- Tre o più di tre

1. Negli scorsi 12 mesi, hai mai avuto rapporti non completi o completi al primo appuntamento (hook-ups)?

- Nessuno
- Uno/Qualche volta
- Spesso

1. Ti è mai stata diagnosticata un’infezione sessualmente trasmissibile (STI)? (Puoi selezionare più di un’opzione)

- No, mai
- Si, mi è stato diagnosticato:
- HIV
- Gonorrea
- Clamidia
- Sifilide
- HPV
- Other: ________

1. Usi le dating-app?

- No, non le ho mai usate
- No, ma le ho usate in passato
- Si, le uso

Domande solo per gli utenti di dating app

1. Da quanti anni usi le dating app?

________

1. Cosa cercavi quando hai installato le dating app?

- Cercavo amici
- Cercavo partner sessuali
- Cercavo partner romantici (es. fidanzato/a)
- Cercavo trasgressione
- Non sapevo cosa stessi cercando

1. Quanto spesso accedi alle dating app?

- Quasi mai
- Una o due volte al mese
- Una o due volte alla settimana
- Una volta al giorno
- Due o tre volte al giorno
- Più di tre volte al giorno

**List of variables**

The table reports the 25 variables that were extracted from the responses to the questionnaire.

**Age =** this is a continuous variable indicating the age of the participant.

**Being_male =** this is a dichotomous variable indicating the assigned-at-birth sex of the participant (1=male, 0= female).

**Being_cisgender =** this is a dichotomous variable indicating the gender of the participant (1=cisgender, 0= other gender).

**Educational_level** = this variable indicates years of education of the participant. The response was coded as follows: 8= “middle school”, 13= “high school”, 16= “triennial degree”, 18= “master degree or more years of education”.

**Being_heterosexual =** this is a dichotomous variable indicating the sexual orientation of the participant (1= heterosexual, 0= non-heterosexual).

**Being_homosexual** = this is a dichotomous variable indicating the sexual orientation of the participant (1= homosexual, 0= non-homosexual).

**Other_sexual_orientation =** this is a dichotomous variable indicating the sexual orientation of the participant (1= other sexual orientations, 0= heterosexual or homosexual).

**Being_single =** this is a dichotomous variable indicating single participants (1=single, 0= in a relationship).

**Being_polyamorous =** this is a dichotomous variable indicating polyamorous participants (1= polyamorous, 0= monogamous).

**Sexual_intercourses =** this categorical variable indicates participants who have had sexual intercourses in their lives (0= not having them, 1= having had only incomplete sexual intercourses, 2= having had complete and incomplete sexual intercourses).

**N_partner_protected** = this continuous variable indicates the number of different partners with whom participants had full protected sexual intercourses last year (0 = none, 1= one or two partners, 2 = three or more partners).

**N_partner_unprotected** = this continuous variable indicates the number of different partners with whom participants had full unprotected sexual intercourses last year (0 = none, 1= one or two partners, 2 = three or more partners).

**Frequency_hook-up =** this continuous variable indicates frequency which participants had full hook-ups last year (0 = never, 1= once or sometimes, 2 = often).

**STI_diagnosis** = this dichotomous variable indicates participants who have had at least one diagnosis of sexually transmitted infection (1= STI diagnosis, 0= no STI diagnosis).

**User_type =** this categorical variable indicates types of dating app users (former user, active user, non-user).

**Being_active_user =** this dichotomous variable indicates active dating app users (1= active users, 0 = other users).

**Being_former_user =** this dichotomous variable indicates former-users (1= former users, 0 = other users).

**Being_non-user =** this dichotomous variable indicates non-users (1= non-users, 0 = other users).

**Usage_years =** this is a continuous variable indicating active users’ years of dating apps utilization.

**Installation_Friends =** this dichotomous variable indicates active users who have installed dating apps to find friends (1= to find friends, 0 = other motivations).

**Romantic_partners =** this dichotomous variable indicates active users who have installed dating apps to find romantic partners (1= to find romantic partners, 0 = other motivations).

**Sexual_partners =** this dichotomous variable indicates active users who have installed dating apps to find sexual partners (1= to find sexual partners, 0 = other motivations).

**Transgression =** this dichotomous variable indicates active users who have installed dating apps to find transgression (1= to find transgression, 0 = other motivations).

**No_motivation =** this dichotomous variable indicates active users who have installed dating apps without a specific motivation (1= no specific motivation, 0 = other motivations).

**App_access_frequency =** this variable indicates how many times active users enter their dating apps. The response was coded as follows: 1= “almost never”, 2 = “once or twice a month”, 3 = “once or twice a week”, 4 = “once a day”, 5 = “two or three times a day”, 6 = “more than 3 times a day”,

**Intermediate steps of the logistic regressions reported in Tables 4, 5, 6, 7 of the main text**

**Table 4**

Model 1 (intercept): *RMSE*=0.767; Model 2 (intercept + being polyamorous): *F-change*_(1, 265)_= 32.214, *p=*3.617e -8, *R^2^=*0.108, *Adjusted R^2^=*0*.*105*, RMSE*=0.726, *ANOVA F*_(1, 265)_= 32.214, *p=*3.617e -8; Model 3 (intercept + being polyamorous + app access frequency): *F-change*_(1, 264)_= 5.944, *p=*0.015, *R^2^=*0.128, *Adjusted R^2^=*0.121*, RMSE*=0.719, *ANOVA F*_(2, 264)_= 19.380, *p=*1.402e -8; Model 4 (intercept + being polyamorous + app access frequency + looking for romantic partners): *F-change*_(1, 263)_= 5.487, *p=*0.020, *R^2^=*0.146, *Adjusted R^2^=*0.136*, RMSE*=0.713, *ANOVA F*_(3, 263)_= 14.968, *p=*5.034e -9; Model 5 (intercept + being polyamorous + app access frequency + looking for romantic partners + looking for friends): *F-change*_(1, 262)_= 6.529, *p=*0.011, *R^2^=*0.167, *Adjusted R^2^=*0.154*, RMSE*=0.706, *ANOVA F*_(4, 262)_= 13.094, *p=*9.767e -10; Model 6 (intercept + being polyamorous + app access frequency + looking for romantic partners + looking for friends + educational level): *F-change*_(1, 261)_= 5.104, *p=*0.025, *R^2^=*0.183, *Adjusted R^2^=*0.167*, RMSE*=0.700, *ANOVA F*_(5, 261)_= 11.660, *p=*3.496e -10.

**Table 5**

Model 1 (intercept): *RMSE*=0.692; Model 2 (intercept + looking for sexual partners): *F-change*_(1, 265)_= 15.007, *p=*1.351e -4, *R^2^=*0.054, *Adjusted R^2^=*0*.*050*, RMSE*=0.675, *ANOVA F*_(1, 265)_= 15.007, *p=*1.351e -4; Model 3 (intercept + looking for sexual partners + usage years): *F-change*_(1, 264)_= 10.314, *p=*0.001, *R^2^=*0.089, *Adjusted R^2^=*0.082*, RMSE*=0.663, *ANOVA F*_(2, 264)_= 12.924, *p=*4.417e -6; Model 4 (intercept + looking for sexual partners + usage years + looking for romantic partners): *F-change*_(1, 263)_= 6.927, *p=*0.009, *R^2^=*0.113, *Adjusted R^2^=*0.102*, RMSE*=0.656, *ANOVA F*_(3, 263)_= 11.118, *p=*6.792e -7; Model 5 (intercept + looking for sexual partners + usage years + looking for romantic partners + being male): *F-change*_(1, 262)_= 4.521, *p=*0.034, *R^2^=*0.128, *Adjusted R^2^=*0.114*, RMSE*=0.652, *ANOVA F*_(4, 262)_= 9.581, *p=*3.032e -7; Model 6 (intercept + looking for sexual partners + usage years + looking for romantic partners + being male + looking for friends): *F-change*_(1, 261)_= 5.774, *p=*0.017, *R^2^=*0.146, *Adjusted R^2^=*0.130*, RMSE*=0.646, *ANOVA F*_(5, 261)_= 8.959, *p=*7.179e -8.

**Table 6**

Model 1 (intercept): *RMSE*=0.792; Model 2 (intercept + app access frequency): *F-change*_(1, 270)_= 33.566, *p=*1.914e -8, *R^2^=*0.111, *Adjusted R^2^=*0*.*107*, RMSE*=0.748, *ANOVA F*_(1, 270)_= 33.566, *p=*1.914e  -8; Model 3 (intercept + app access frequency + looking for sexual partners): *F-change*_(1, 269)_= 23.769, *p=*1.859e -6, *R^2^=*0.183, *Adjusted R^2^=*0.177*, RMSE*=0.719, *ANOVA F*_(2, 269)_= 30.083, *p=*1.620e -12; Model 4 (intercept + app access frequency + looking for sexual partners + being heterosexual): *F-change*_(1, 268)_= 9.406, *p=*0.002, *R^2^=*0.210, *Adjusted R^2^=*0.202*, RMSE*=0.708, *ANOVA F*_(3, 268)_= 23.817, *p=*1.073e -13; Model 5 (intercept + app access frequency + looking for sexual partners + being heterosexual + being polyamorous): *F-change*_(1, 267)_= 4.900, *p=*0.028, *R^2^=*0.225, *Adjusted R^2^=*0.213*, RMSE*=0.702, *ANOVA F*_(4, 267)_= 19.348, *p=*5.421e -14.

**Table 7**

Model 1 (intercept): df=271; Model 2 (intercept + usage years): df=270, Δχ2=32.434, p=1.233e -8, R² = 0.114 (McFadden), 0.173 (Nagelkerke), 0.129 (Tjur), 0.112 (Cox & Snell); Model 3 (intercept + usage years + app access frequency): df=269, Δχ2=20.474, p=6.046e -6, R² = 0.186 (McFadden), 0.273 (Nagelkerke), 0.212 (Tjur), 0.177 (Cox & Snell); Model 4 (intercept + usage years + app access frequency + being heterosexual): df=268, Δχ2=6.660, p=0.010, R² = 0.209 (McFadden), 0.303 (Nagelkerke), 0.239 (Tjur), 0.197 (Cox & Snell); Model 5 (intercept + usage years + app access frequency + being heterosexual + age): df=267, Δχ2=5.115, p=0.024, R² = 0.227 (McFadden), 0.326 (Nagelkerke), 0.252 (Tjur), 0.212 (Cox & Snell); Model 6 (intercept + usage years + app access frequency + being heterosexual + age + no specific motivation): df=266, Δχ2=4.151, p=0.042, R² = 0.242 (McFadden), 0.345 (Nagelkerke), 0.265 (Tjur), 0.224 (Cox & Snell).
